# OsJAB1 Positively Regulates Ascorbate Biosynthesis and Negatively Regulates Salt Tolerance Due to Inhibiting Early-Stage Salt-Induced ROS Accumulation in Rice

**DOI:** 10.3390/plants12223859

**Published:** 2023-11-15

**Authors:** Jiayi Wang, Chuanyu Zhang, Hua Li, Yuejun Xu, Bo Zhang, Fuyu Zheng, Beiping Zhao, Haiwen Zhang, Hui Zhao, Baohai Liu, Minggang Xiao, Zhijin Zhang

**Affiliations:** 1 Biotechnology Research Institute, Chinese Academy of Agricultural Sciences, Beijing 100081, China; 15524115399@163.com (J.W.); zcy198610@163.com (C.Z.); lihua2436@163.com (H.L.); 18262878125@163.com (Y.X.); zhanghaiwen@caas.cn (H.Z.); 2 National Key Facility of Crop Gene Resources and Genetic Improvement, Sanya 571763, China; 3 Biotechnology Research Institute, Heilongjiang Academy of Agricultural Sciences, Harbin 150028, China; zhangbo222@sina.com (B.Z.); zhengfy1010@163.com (F.Z.); 13945094556@139.com (B.Z.); shslbh@163.com (B.L.); 4 Institute of Tropical Bioscience and Biotechnology, Chinese Academy of Tropical Agricultural Sciences, Haikou 571101, China; zhaohui_hbu@126.com

**Keywords:** salt stress, OsJAB1, ascorbate, ROS, rice

## Abstract

Reactive oxygen species (ROS) play dual roles in plant stress response, but how plants modulate the dual roles of ROS in stress response is still obscure. *OsJAB1* (*JUN-activation-domain-binding protein 1*) encodes the rice CSN5 (COP9 signalsome subunit 5). This study showed that, similar to the *Arabidopsis* homolog gene *CSN5B*, *OsJAB1*-overexpressing (driven by a CaMV 35S promoter) plants (OEs) impaired rice salt stress tolerance; in contrast, *OsJAB1*-inhibited-expression (using RNA-interfering technology) plants (RIs) enhanced rice salt stress tolerance. Differing from CSN5B that negatively regulated ascorbate (Asc) biosynthesis, Asc content increased in OEs and decreased in RIs. ROS analysis showed that RIs clearly increased, but OEs inhibited ROS accumulation at the early stage of salt treatment; in contrast, RIs clearly decreased, but OEs promoted ROS accumulation at the late stage of salt treatment. The qPCR revealed that OEs decreased but RIs enhanced the expressions of ROS-scavenging genes. This indicated that OsJAB1 negatively regulated rice salt stress tolerance by suppressing the expression of ROS-scavenging genes. This study provided new insights into the CSN5 homologous protein named OsJAB1 in rice, which developed different functions during long-term evolution. How OsJAB1 regulates the Asc biosynthesis that coordinates the balance between cell redox signaling and ROS scavenging needs to be investigated in the future.

## 1. Introduction

Environment change is one of the important factors which affects soil properties and plant development. The increased salinization of soil will lead to a significant decrease in crop fields [1]. Salinity stress has multiple adverse effects on the growth and development of crops. However, plants can adapt to stress conditions by regulating their many morphological, cellular, anatomical, and physiological changes and endogenous hormone levels [2]. One of the main mechanisms for high salinity-induced damage is the accumulation of reactive oxygen species (ROS) [3]. ROS play dual roles in the response of plants to adverse conditions. On the one hand, low levels of accumulated ROS can activate multiple stress-responsive pathways in plants in order to adapt to adverse conditions; on the other hand, high levels of accumulated ROS seriously damage plant cell composition and disrupt plant growth and development processes [4,5]. However, there are few studies detailing how plants regulate the relationship between ROS signaling and damage. 

ROS, such as hydrogen peroxide (H_2_O_2_) and superoxide anion radical (O_2_^−^), can be produced and can accumulate in plants exposed to abiotic stresses [6,7]. The accumulated ROS are harmful for plants; therefore, plants have developed an effective ROS-scavenging system to inhibit the accumulation of stress-induced ROS in order to adapt to adverse conditions [8,9]. Antioxidants, such as ascorbate (Asc), are essential for ROS-scavenging in a mechanism called the Mehler reaction [10,11]. The Mehler reaction is related to abiotic stress, for instance, exposure to light, cold, drought and salt stress [11]. De novo synthesis or recycle regeneration of Asc can help plants scavenge ROS and enhance salt stress tolerance [10,11]. Asc can be regenerated from monodehydroascorbate (MDHA) and dehydroascorbate (DHA) with MDHA reductases (MDHARs) and DHA reductases (DHARs), respectively. Through these, more Asc can be produced to help plants protect cells against ROS damage [11]. For example, overexpressing *AeMDHAR* (from the mangrove plant Acanthus ebracteatus) in rice showed that MDHAR had a higher enzyme activity under salt treatment. All overexpressing transgenic lines showed tolerance to salt stress and had better yields compared to WT [12]. Asc may play complex roles in the responses of plants to condition stresses. For example, in *Arabidopsis*, exogenous Asc could enhance the salt stress of salt-sensitive *ABI4*-overexpressing *(ABA INSENSITIVE* 4, *ABI4*) plants. *ABI4*-overexpressing plants decreased their salt tolerance as increasing Asc content inhibited ROS signaling. In contrast, *ABI4* loss-of-function mutants enhanced plant salt stress tolerance due to inhibited Asc biosynthesis activating ROS signaling [13]. 

The photomorphogenic factor COP9 signalosome (CSN) has eight subunits (CSN1~8) [14]. These subunits play unique and critical roles in regulating plant growth and developmental processes and in the responsive system to adverse environment conditions [14]. Studies have shown that the CSN5 subunit of COP9 can bind numerous regulators and regulate their stability, resulting in multiple functions of CSN5 [15]. In *Arabidopsis*, CSN5 is encoded by two homologous genes *CSN5A* and *CSN5B*. The *csn5a* mutant displayed several plant phenotypes, but *csn5b* had less effect on plant phenotype [16]. However, CSN5B can interact with GDP-mannose pyrophosphorylase (VTC1), a key Asc biosynthesis enzyme, and accelerate the degradation of the VTC1 protein, inhibiting Asc biosynthesis. Thus, *CSN5B*-overexpressing plants have lower Asc content and impair salt stress tolerance [17]. CSN5B can also interact with a C2H2 zinc-finger protein SlZF3 from tomato (*Solanum lycopersicum*), which can compete against VTC1, so overexpressing *SlZF3* can protect VTC1 from CSN5B mediated degradation and promote Asc accumulation to scavenge ROS induced by salt stress [18], which ultimately enhances plant salt stress tolerance. Therefore, CSN5B can regulate plant tolerance to salt stress through the pathway of Asc biosynthesis.

Rice is one of the most important crops in the world. Differing from *Arabidopsis*, the rice COP9 signalosome only has one CSN5 subunit OsJAB1 (JUN-activation-domain-binding protein 1) [19]. Although studies have shown that the CSN5 subunit plays a crucial role in the responses of multiple plants to adverse stresses [17,20,21], the functions of OsJAB1 in response to condition stresses are still unclear. This study revealed that, in contrast to *Arabidopsis* CSN5B, OsJAB1 can inhibit ROS signaling and impair rice salt stress tolerance. In addition, we identified the regulatory relationship between ROS signaling and accumulation during the adaption of plants to salt stress.

## 2. Results

### 2.1. The Expression of OsJAB1 (JUN-Activation-Domain-Binding Protein 1) Is Induced by Salt Stress

In contrast to *Arabidopsis CSN5*, which has two homologous genes (*CSN5A* and *CSN5B*), rice had only one *CSN5* homologous gene, *OsJAB1* (Figure S1). The homology of the amino acids of CSN5A and CSN5B was about 77.6% (Figure S1).

**Figure 1:**
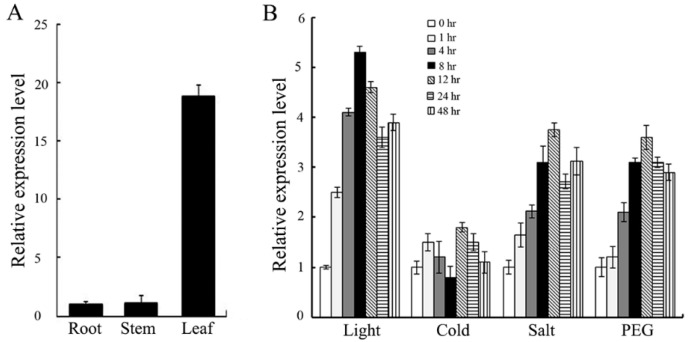
Transcript levels of *OsJAB1* in different organs and under different abiotic stresses. (**A**) Expression levels of *OsJAB1* in rice roots, stems and leaves. (**B**) The expression levels of *OsJAB1* under high light, cold, salt and PEG treatments. The expression levels of *OsJAB1* under normal conditions (0 h) were set to a value of 1. Graph shows the relative expression levels of *OsJAB1* under abiotic stress treatment vs. normal conditions and presents the average from three independent experiments. Bars represent the standard error (±SE).

To analyze the function of OsJAB1 in the rice salinity stress response, firstly, we studied the expression pattern of *OsJAB1* in different organs under different treatments. The qPCR results showed that the expression of *OsJAB1* was high in leaves but weak in roots and stems, revealing that *OsJAB1* predominantly expressed in rice leaves (Figure 1A). Next, we analyzed the expression pattern of *OsJAB1* under different treatments. The qPCR results revealed that light, salt and drought (PEG) treatments could induce *OsJAB1* expression (Figure 1B). Strong light treatment quickly induced *OsJAB1* expression in 1 h; in contrast, salt and drought treatment clearly enhanced *OsJAB1* expression in 4 h (Figure 1B). These results indicated that OsJAB1, similar to *Arabidopsis* CSN5B, might be involved in rice salt stress responses.

### 2.2. OsJAB1 Negatively Regulates Rice Salt Stress Tolerance

**Figure 2:**
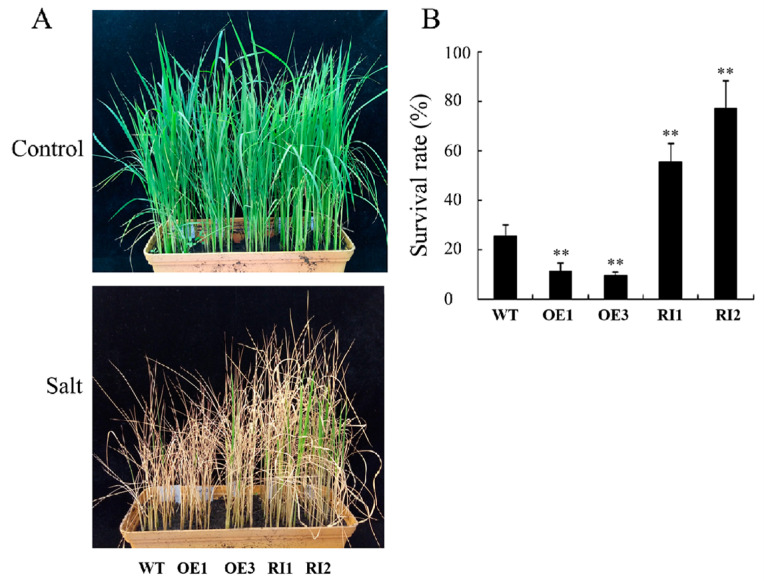
OsJAB1 negatively regulates rice salinity stress tolerance. (**A**) Phenotype of rice seedlings under salt stress in soil. Three-week-old rice seedlings grown in soil were treated with 150 mM NaCl for 7 days followed by a 7-day recovery. (**B**) Survival rate of rice seedlings treated with 150 mM NaCl for 7 days followed by a 7-day recovery. Experiments were repeated at least three times. Bars represent the SE (±), and asterisks indicate a significant difference. Significance was evaluated using the *t*-test (** *p* < 0.01).

To further analyze the role of OsJAB1 in the rice salinity stress response, we produced *OsJAB1*-overexpressing transgenic plants (OEs) and RNA-interfering transgenic plants (RIs) in Zhonghua 11 (WT) backgrounds (Figures S2 and S3). After a 7-day treatment with 150 mM NaCl and a 7-day recovery, the OEs showed a salt-stress-sensitive phenotype (Figure 2A). The survival rate of the OEs was about 15%; in contrast, the survival rate of the WT was about 25% (Figure 2B). The RIs demonstrated a salinity-stress-tolerant phenotype (Figure 2A). The survival rate of RIs was about 55~75% (Figure 2B). These results indicated that OsJAB1, similar to *Arabidopsis* CSN5B, negatively regulated rice salinity stress tolerance.

### 2.3. OsJAB1 Positively Regulates Rice Asc Biosynthesis

**Figure 3:**
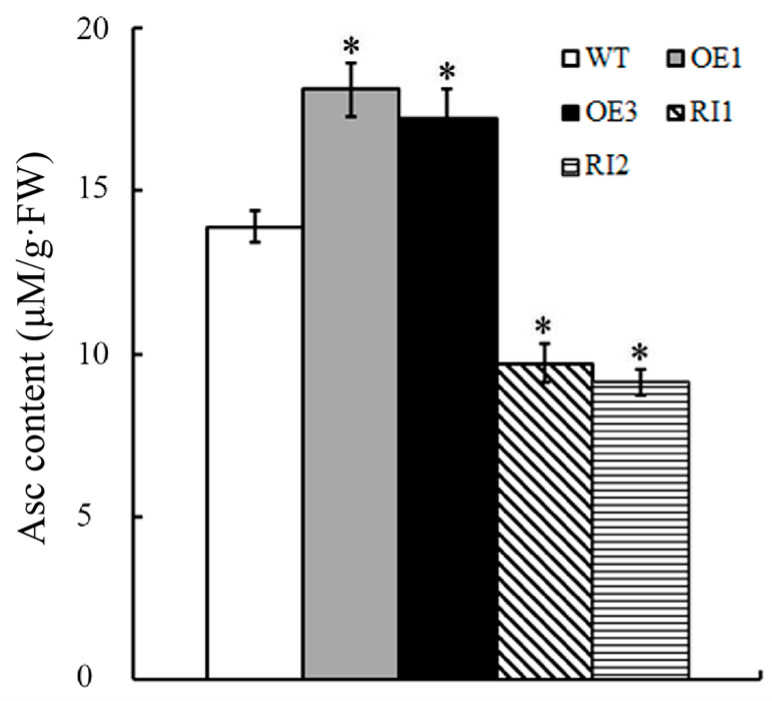
The effects of OsJAB1on rice ascorbate biosynthesis. The Asc content of four-week-old *OsJAB1* transgenic seedlings grown in soil treated with 150 mM NaCl for 7 days was measured. Graph presents the average of three repeated experiments. Bars represent the SE (±), and asterisks indicate significant differences. Significance was evaluated using the *t*-test (* *p* < 0.05).

A previous study showed that *Arabidopsis* CSN5B impaired plant salinity stress tolerance by inhibiting Asc biosynthesis [13]. To confirm that OsJAB1 regulated the rice salinity stress response using the same mechanism as CSN5B, we analyzed the effect of OsJAB1 on Asc biosynthesis. The results from the Asc content measurement revealed that the Asc content in the OEs (about 18.1 μM/g·FW and 17.2 μM/g·FW in transgenic lines OE1 and OE3, respectively) was clearly higher than that in the WT (about 13.9 μM/g·FW). In contrast, the Asc content in the RIs (about 9.7 μM/g·FW and 9.1 μM/g·FW in transgenic lines RI1 and RI2, respectively) was significantly less than that in the WT (Figure 3). These results revealed that, in contrast to *Arabidopsis* CSN5B, which negatively regulated Asc biosynthesis, OsJAB1 positively regulated Asc biosynthesis, indicating that OsJAB1 might be involved in the salt stress response of rice through a pathway different from the one used by CSN5B in *Arabidopsis*.

### 2.4. OsJAB1 Affects the Accumulation of Salt-Stress-Induced ROS

**Figure 4:**
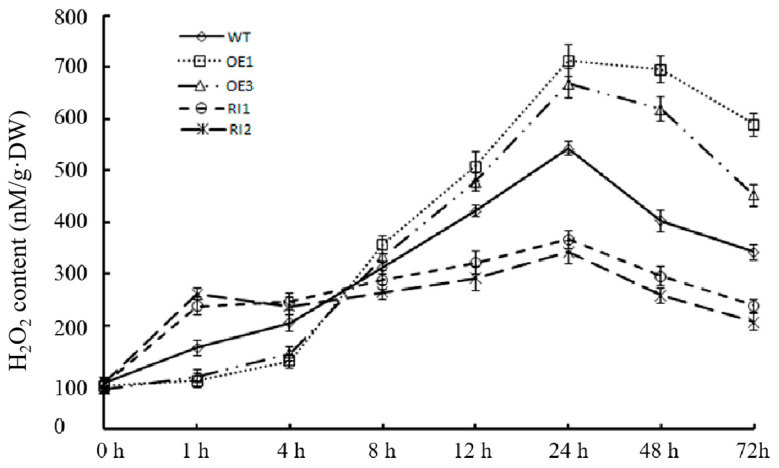
The effect of OsJAB1 on H_2_O_2_ accumulation in rice leaves under salinity stress. Three-week-old *OsJAB1* transgenic seedlings grown in soil were used to analyze the effect of OsJAB1 on H_2_O_2_ accumulation in rice leaves at various time points during the 150 mM NaCl treatment. Bars represent the SE (±) from three experiments.

Considering the important role of ROS levels in plant stress response and the possibility that Asc could regulate ROS levels in plants, we studied the effects of OsJAB1 on rice ROS accumulation under salinity stress. H_2_O_2_ is an important component of ROS. To analyze the effect of OsJAB1 on rice ROS accumulation under salinity stress, we measured the H_2_O_2_ content of four-week-old WT, OEs and RIs at various time points after 150 mM NaCl treatment. The results showed that H_2_O_2_ accumulated more quickly in RIs but more slowly in OEs than in the WT in the early stage of the salt treatment (Figure 4). After 1 h of salt treatment, the levels of H_2_O_2_ in the OEs were significantly lower compared to those in the RIs and WT plants: the H_2_O_2_ content increased by 94 nM/g·DW and 103 nM/g·DW in RI1 and RI2, respectively, and the H_2_O_2_ content in the WT increased by 69 nM/g·DW; in contrast, the H_2_O_2_ content only increased by 23 nM/g·DW and 26 nM/g·DW in OE1 and OE3, respectively (Figure 4). However, during the late stage of the salt treatment, the RIs maintained the H_2_O_2_ content at a relatively low level, about 400 nM/g·DW, but the OEs accumulated more H_2_O_2_, which amounted to over 750 nM/g·DW at the late stage of the salt treatment (Figure 4). These results indicated that OsJAB1 enhanced the H_2_O_2_ scavenging ability of plants and inhibited ROS accumulation in the early stage of the salt stress treatment, impaired H_2_O_2_ scavenging ability in the late stage of the salt stress treatment, and negatively regulated salt tolerance.

### 2.5. OsJAB1 Impaired the Expression of ROS-Scavenging Genes

**Figure 5:**
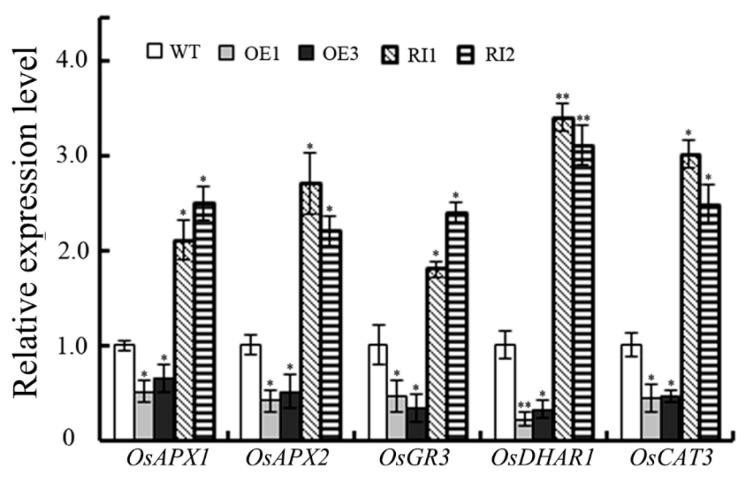
Expression levels of ROS-scavenging-related genes in *OsJAB1* transgenic plants under salinity stress. Four-week-old rice seedlings were used to analyze the relative expression levels of ROS-scavenging genes after a 2-day high salinity treatment with 150 mM NaCl. The expression of *OsActin1* was used as an internal control. The graph presents the relative expression levels of genes compared to those in WT. Bars represent the SE (±) from three experiments. Significance was evaluated using the *t*-test (* *p* < 0.05 and ** *p* < 0.01).

The ability of plants to scavenge ROS is closely related to the biosynthesis of antioxidants and the activity of a ROS-scavenging enzyme. To determine how OsJAB1 positively regulated antioxidant Asc biosynthesis yet decreased the ROS-scavenging ability and salt stress tolerance of rice, we analyzed the expression of ROS-scavenging enzyme genes [19,20,21,22,23,24]. The results showed that the expression of ROS-scavenging enzyme genes, such as the cytosolic ascorbate peroxidase (APX) genes *OsAPX1* and *OsAPX2* and the catalase (CAT) gene *OsCAT3*, increased and decreased in RIs and OEs, respectively, after a 2-day salinity treatment (Figure 5). In addition to ROS-scavenging enzyme genes, the expression of Asc regeneration genes, such as the glutathione reductase (GR) gene *OsGR3* and the dehydroascorbate reductase (DHAR) gene *DHAR1*, also increased and decreased in RIs and OEs, respectively, after a 2-day salt treatment (Figure 5). Since these ROS-scavenging enzyme genes and Asc regeneration genes play important roles in rice abiotic stress tolerance [22,23,24,25,26,27,28,29], impaired OsJAB1 function in RIs enhanced their ROS-scavenging ability and rice salt stress tolerance due to increased expressions of ROS-scavenging enzyme genes compared to the WT and OEs.

## 3. Discussion

ROS play a critical role in the response of plants to salt stress [3]. ROS have two roles in the response of plants to adverse conditions: damaging plant cells and organs and activating stress response signaling to enhance plant environment stress tolerance [4,5]. However, how plants modulate the ROS signal pathway in response to salt stress has not been well demonstrated. Here, we found the detailed relationships that rice modulates between ROS signaling and ROS scavenging, which will help people study how to enhance plant salt stress tolerance using biotechnology.

Previous studies showed that the CSN5 subunit of the COP9 complex negatively regulated plant abiotic stress tolerance. For example, in *Arabidopsis*, *CSN5B*-overexpressing plants showed a salinity-sensitive phenotype, but loss of function in the *CSN5B* mutant enhanced salt tolerance [17]. Tomato Cys2/His2-type zinc-finger protein SlZF3 can positively regulate plant salt tolerance by inhibiting CSN5B-mediated VTC1 degradation in tomato and *Arabidopsis* [18]. Similar to *CSN5B*, the overexpression of *OsJAB1* impaired salinity stress tolerance while depressing *OsJAB1*-expression-enhanced tolerance (Figure 2), indicating that OsJAB1 and its homology have similar physiological functions under salt stress. However, in contrast to CSN5B inhibiting Asc biosynthesis in tomato and *Arabidopsis*, overexpressing *OsJAB1* increased rice Asc content, and impairing the OsJAB1 function inhibited Asc biosynthesis (Figure 3), revealing that OsJAB1 positively regulates Asc biosynthesis. Though several studies revealed that CSN5B and its homology negatively regulated Asc biosynthesis. Such as, in apple, the MdAMR1L1 protein can interact with the Asc biosynthesis key enzyme MdGMP1 and promote its degradation in order to inhibit Asc synthesis [30]. In *Arabidopsis*, SRAS1 (Salt-Responsive Alternatively Spliced gene 1) promotes the degradation of CSN5A and enhances plant salt stress tolerance [21]. CSN5 plays multiple roles in plants except for promoting protein degradation [31]. Moreover, *Arabidopsis* has two CSN5 homology proteins, CSN5A and CSN5B, which display different functions and are involved in the physiology process [16]. CSN5A plays an important role in plant growth and development, and CSN5B is involved in the response to environment stress [13,18,21], but rice CSN5 is only encoded by *OsJAB1*, which also indicates that CSN5 may have functional differences between plant species. Our results revealed that OsJAB1 might be involved in depressing the function of a negative regulator of Asc biosynthesis or in enhancing the stability of an Asc biosynthesis key enzyme and Asc accumulation, which was different from CSN5 that negatively regulated Asc biosynthesis by promoting the degradation of the Asc biosynthesis key enzyme VTC1.

ROS play an important role in the adaption of plants to adverse conditions. Cell redox state has a profound effect on the adaption of plants to salinity stress and is closely related to the activity of a large number of physiological and biochemical processes in plants [32,33,34]. ROS have two different functions in plant stress response. High levels of accumulated ROS result in oxidative damage and impaired plant stress tolerance, and low levels of accumulated ROS can act as a stress signal molecule to activate multiple stress-responsive plant pathways and enhance plant stress tolerance [4,5].

Asc plays critical roles in regulating plant abiotic tolerance by modulating ROS scavenging to affect cell redox state and stress signaling as an antioxidant [35]. Asc can scavenge accumulated ROS, protecting plants from oxidative damage and enhancing stress tolerance [34,36]. For example, in *Arabidopsis*, enhancing Asc biosynthesis can help plants scavenge more ROS and significantly improve their salinity stress tolerance [37,38,39]. Disrupting de novo Asc synthesis significantly decreases plant stress tolerance [40]. In rice, the overexpression of *OsVTC1* increased Asc content and enhanced salt tolerance; in contrast, decreasing *OsVTC1* expression clearly impaired salt tolerance in rice [38,41]. In addition to de novo synthesis, the regeneration of Asc is also critical for plants in preventing ROS damage and enhancing abiotic stress tolerance [10,24,42]. Asc recycle enzymes, such as monodehydroascorbate reductase (MDAR) and DHAR, can effectively regenerate Asc to scavenge ROS and enhance plant stress tolerance [40,42,43]. For example, the overexpression of *DHAR* in chloroplasts can clearly enhance the chloroplasts’ ROS-scavenging capacity and improve the abiotic stress tolerance of transgenic tobacco, rice and *Arabidopsis* [44]. In addition, the mutation of cytosolic *DHAR* (*cytDHAR*) decreases oxidative stress tolerance in *Arabidopsis* [45].

**Figure 6:**
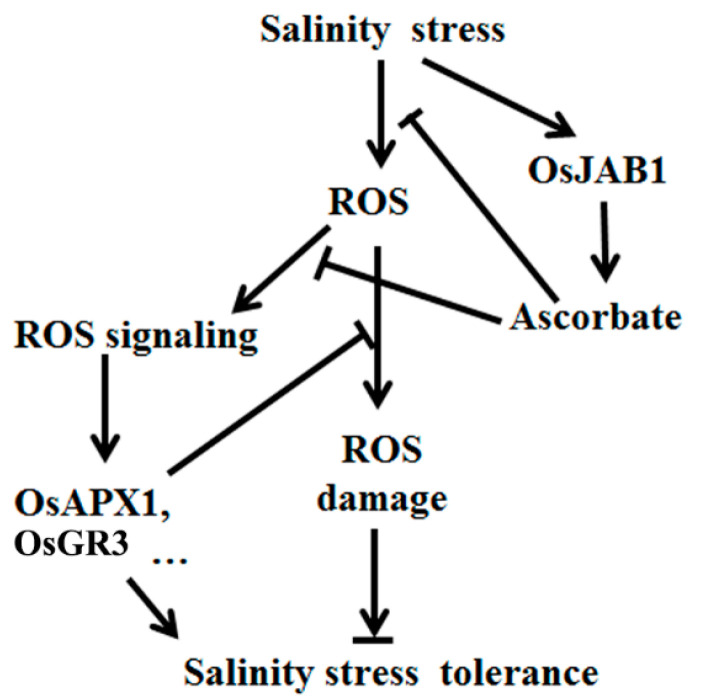
Ascorbate (Asc) plays dual roles in the response of plants to salinity. On the one hand, Asc can scavenge ROS, protect plants from oxidative damage and enhance salinity stress tolerance. On the other hand, Asc inhibits ROS signaling. Under salt stress, overexpression of *OsJAB1* increases the ascorbate content in rice, which can scavenge more ROS and delay ROS accumulation, in turn inhibiting the salt stress response of rice through the ROS signaling pathway that activates the expression of ROS-scavenging genes and impairs salinity stress tolerance. In contrast, impairing the expression of OsJAB1 helps rice quickly accumulate ROS, which can effectively activate the rice ROS-scavenging system and enhance rice salinity stress tolerance.

Moreover, as well as directly scavenging ROS, Asc is also involved in ROS signal transduction through ROS homeostasis. The enzymes that are involved in Asc metabolism and regeneration are also critical for regulating cell redox signals [46,47]. Disrupting the action of the *Arabidopsis* APX6 function reduces seed Asc regeneration, accumulates DHA disrupting cell redox homeostasis and modulates ROS signaling, which results in high levels of ROS and inhibited plant stress tolerance [11,48]. Asc oxidases (AOs) are additional important Asc oxidization enzymes, which are also involved in cellular signal transduction [49,50]. Apoplast DHA from the oxidation of Asc by AOs can be quickly transported into the cytoplasm and quickly activates plant stress response in time [51,52]. Gain-of-function mutations in AOs block the cellular redox flux and disrupt the signaling pathways induced by environmental stress [49,53]. In stomata, DHARs are crucial for Asc regeneration from DHA, which is very important to transmitting stress signals [11]. Overexpression of stomata-located DHARs cause Asc reduction, inhibit guard cell response to ABA and H_2_O_2_ signaling, and result in the stomata remaining open and losing more water, further resulting in decreased plant drought tolerance in tobacco. In contrast, suppressing the expression of these *DHARs* promotes Asc production, accumulates more H_2_O_2_—which activates the ABA and H_2_O_2_ signaling pathways—and accelerates stomata closure, reducing water loss and enhancing plant drought tolerance [54]. Overexpressing *OsJAB1* retarded ROS accumulation at the early stage, impaired the expression of ROS-scavenging genes and decreased rice ROS scavenging and salinity stress tolerance at the late stage of the high salinity stress treatment (Figure 2, Figure 4 and Figure 5). Suppressing the expression of *OsJAB1* accelerated ROS accumulation at the early stage, increased the expression of ROS scavenging genes and enhanced rice ROS scavenging and salinity stress tolerance at the late stage during the high salinity stress treatment (Figure 4, Figure 5 and Figure 6). Therefore, the overexpression of *OsJAB1* impaired the expressions of ROS-scavenging genes and decreased ROS-scavenging ability, and impairing *OsJAB1* function increased the expressions of ROS-scavenging genes and enhanced ROS scavenging ability (Figure 4 and Figure 5), indicating that OsJAB1 regulated Asc synthesis through affecting ROS signaling [11]. Xiao et al. reviewed the literature, finding that Asc can scavenge ROS and be useful for plant stress tolerance as an antioxidant; however, in some cases, increasing amounts of Asc may inhibit Asc biosynthesis and regeneration, which can regulate ROS signaling and impair plant stress tolerance [11]. Thus, OsJAB1 may be involved in a different mechanism responding to salt stress. On one hand, increasing Asc content results in suppressing Asc regeneration and plant stress tolerance; on the other hand, OsJAB1 may have higher inhibition for expressions of ROS-scavenging genes, subsequently promoting Asc biosynthesis. In further studies, we may investigate the relationship between increasing Asc content and inhibition of ROS-scavenging ability.

Altogether, this study shows that rice OsJAB1 negatively regulates rice salinity stress response by disrupting ROS signaling (Figure 6), which is different from the mechanism of *Arabidopsis* homolog CSN5B that negatively regulates *Arabidopsis* salinity stress response by limiting antioxidant Asc biosynthesis, indicating that different plant species develop different stress response mechanisms during long-term evolution.

## 4. Materials and Methods

### 4.1. Plant Materials and Growth Condition

The seeds of different rice materials were germinated at 37 °C for 2 days and then grown in a greenhouse at 27–30 °C (16 h light period) and 25 °C (8 h dark period). *Japonica* cultivar ZH11 (*Oryza sativa* L. ssp. *japonica* cv. Zhonghua 11) as wild-type (WT), independent homozygous *OsJAB1* T3 overexpression plants (OE1 and OE3 lines) and RNA-interfering plants (RI1 and RI2 lines) were used in this study. To examine the role of *OsJAB1* in different organs, transgenic plants and, under salt stress treatment, one-week-old WT and transgenic seedlings were transferred into a small concrete pool under normal conditions and grown for another two weeks before the salt treatment. For analyzing the expression level of *OsJAB1* under different treatments, three-week-old plants grown in soil were used. For light treatment, plants were treated with continuous lighting for 48 h. For cold treatment, plants were treated under 4 °C for according time points. For salt treatment, plants were treated with 150 mM NaCl for according time points. For PEG treatment, plants were treated with 250 mM PEG for according time points.

### 4.2. The Generation of Transgenic Rice

To generate *OsJAB1* RNAi plants, the sequence of the *OsJAB1* CDS was designed as the target sequence. The specific target sequence was cloned using the primers 5′-ATCTCCTCGAGGCGCTCCTCAAGATGGTC-3′ and 5′-ATCTCAGATCTGCATCTGAGTAGACACAT-3′ and, then, introduced into vector pUCCRNAi using XhoI and BglII sites. The constructed pUCCRNAi vector was digested with SalI and BamHI. Following this, the digested DNA fragment was linked with the digested vector with XhoI and BglII to obtain the DNA fragments that contained the forward and reverse targeted sequences, which were further cloned into the plant vector pCAMBIA2300 by digestion at the PstI site. Then, the resulting plasmid was introduced into *Agrobacterium* and subsequently transformed into ZH11 using Agrobacterium-mediated transformation. The transformed plants were selected using G418. Homozygous independent *OsJAB1* RNA interference (RNAi) transgenic lines RI1 and RI2 were used in the present study. To produce *OsJAB1* overexpressing transgenic plants, full-length ORF of *OsJAB1* was cloned using the primers 5’-ATCTCACTAGTATGGAGCCCACCTCGTCG-3’ and 5′-ATCTCGTCGACTCATGCTTCAACCATAGGC-3’ and, then, introduced into plant expression vector pCAMBIA 1307 using SpeI and SalI. The constructed vector was introduced into *Agrobacterium* and subsequently transformed into rice ZH11 using *Agrobacterium*-mediated transformation. The *OsJAB1*-overexpressing transgenic lines were selected with hygromycin and confirmed with qPCR. Homozygous independent *OsJAB1*-overexpressing T3 transgenic lines OE1 and OE3 were used in the present study. The expression level of *OsJAB1* in the transgenic lines was analyzed with qPCR using the primers listed in Table S1. The detailed methods were described in a previous study [41].

### 4.3. Analysis of Salt Stress Tolerance

To examine the salt tolerance of the transgenic lines, three-week-old seedlings of OEs and RIs were treated with a 150 mM NaCl solution for 7 days, followed by watering without NaCl for an additional 7 days. Subsequently, the percentage survival rate of rice seedlings was determined (plants with green leaves represented surviving seedlings).

### 4.4. Quantitative Real-Time PCR (qPCR) Assay

To examine the expression of *OsJAB1* under different stress treatments, four-week-old ZH11 seedlings were grown at 4 °C or sprayed with 150 mM NaCl 10% (*w*/*v*) PEG6000 solution. The seedlings were harvested at different time points and subsequently stored in liquid nitrogen for RNA extraction. Total RNA was isolated from the WT, OEs and RIs to confirm the expression levels of *OsJAB1* using TRIzol solution (Tiangen, Beijing, China). Approximately 2 μg of total RNA was reverse transcribed using oligo (dT) primers and M-MLV reverse transcriptase, according to the manufacturer’s instructions (Toyobo, Osaka, Japan). qPCR was performed using the SYBR Green Mix (Takara, Dalian, China, Cat No. 330523) and iQ5, according to the manufacturer’s instructions (Bio-Rad iQ5, Hercules, CA, USA). In the analysis of expression patterns of *OsJAB1* in different organs, the transcript level of *OsJAB1* in root was assigned a value of 1, and the results of *OsJAB1* expression in other organs are shown in the figure as the relative expression levels compared to the roots. In the analysis of expression patterns of *OsJAB1* under abiotic stresses, the transcript level of *OsJAB1* under control condition (0 h) was assigned a value of 1, and the results shown in the figure are the relative expression levels of *OsJAB1* at other time points compared to 0 h. In the analysis of expression patterns of *OsJAB1* in OEs and RIs, the transcript levels of *OsJAB1* in ZH11 were assigned a value of 1, and the results shown in the figure are the relative expression levels compared to that of ZH11. The qPCR reactions were performed in biological triplicates for each individual line, and threshold cycle values were quantified using the relative quantification method. The expression of the *OsActin1* gene was used as an internal standard, and the relative expression values of *OsJAB1* were calculated using the comparative Ct method as described previously by Qin [41]. The primers used in qPCR assay are listed in Table S1.

### 4.5. Asc Content Measurement

To measure the Asc content, four-week-old rice seedlings were harvested and stored in liquid nitrogen. An approximately 0.2 g sample (fresh weight) was ground into a fine powder in liquid nitrogen with a mortar and pestle. The extraction and measurement of the Asc were performed according to methods described by Qin [41].

### 4.6. H_2_O_2_ Content Measurement

The H_2_O_2_ content was analyzed using the FOX1 method [55]. To measure Asc content, fifty-milligram fresh tissue from three-week-old plants was homogenized in 200 mM perchloric acid (HClO_4_). After centrifugation at 4 °C, 10,000× *g* for 15 min, 500 μL of supernatant was transferred to a 1.5 mL tube. A 100 μL volume of supernatant was used to measure the H_2_O_2_ content, according to the method of Mei [55]. The specificity for H_2_O_2_ was tested by eliminating H_2_O_2_ in the reaction mixture with catalase (CAT). Standard curves for H_2_O_2_ were obtained for each independent experiment by adding variable amounts of H_2_O_2._

### 4.7. Statistical Analysis

To analyze the differences among different plant materials under salt stress, 40–50 seedlings of the WT, OEs and RIs were treated under the same growing conditions. To analyze the differences among different materials in Asc and H_2_O_2_ content and gene expression level, each sample was obtained from three to five different seedlings of the WT, OE and RI lines. All above experiments were repeated three times, and the significant differences were evaluated using *t*-tests (* *p* < 0.05 and ** *p* < 0.01).

## 5. Conclusions

The COP9 signalosome CSN5 subunit plays an important role in the responses of multiple plants to adverse stresses. In contrast to *Arabidopsis* homolog CSN5B, which inhibits antioxidant Asc biosynthesis and decreases plant salinity stress tolerance, OsJAB1 increases Asc content but negatively regulates rice salt tolerance. OsJAB1 negatively regulates rice salinity stress tolerance through inhibiting Asc regeneration; OsJAB1 also disrupts ROS accumulation at the early stage of salt stress treatment and inhibits ROS signaling, which impairs the expressions of ROS-scavenging genes under salinity stress. Thus, OsJAB1 positively regulates ascorbate biosynthesis, which inhibits ROS accumulation at the early stage of salt treatment and inhibits salt-stress-responding ROS signaling; signal inhibition, in turn, impairs the expression of the ROS-scavenging genes activated by the salt stress-responsive ROS signal and decreases rice salt stress tolerance.

## Data Availability

All the data in this study are available within this article and its Supplementary Information Files.

## Supplementary Materials

The following supporting information can be downloaded at: https://www.mdpi.com/article/10.3390/plants12223859/s1. Figure S1: Phylogenetic analysis of *Arabidopsis* CSN5B homologous proteins and alignment of OsJAB1 and CSN5B. (A) Phylogenetic analysis of OsJAB1 homologous proteins in different plant species. The phylogeny was analyzed using the Blastp search program of the National Center for Biotechnology Information (NCBI, http://www.ncbi.nlm.nih.gov/, accessed on 13 March 2021). (B) The alignment of OsJAB1 and CSN5B proteins and different colors represent different similarity. Figure S2: The expression levels of *OsJAB1* in four-week-old seedlings of OEs compared to those in WT. The expression level of *OsActin1* was used as an internal control. Bars represent the SE (±) from three repeated experiments. Asterisks show the significant differences evaluated using *t*-tests (** *p* < 0.01). Figure S3: The expression levels of *OsJAB1* in four-week-old seedlings of RIs compared to those in WT. The expression of *OsActin1* was used as an internal control. Bars represent the SE (±) from three repeated experiments. Asterisks represent the significant differences as evaluated using *t*-tests (* *p* < 0.05 and ** *p* < 0.01). Table S1: Primers used in quantitative real-time PCR.
