# Supplementary material for: OsJAB1 Positively Regulates Ascorbate Biosynthesis and Negatively Regulates Salt Tolerance Due to Inhibiting Early-Stage Salt-Induced ROS Accumulation in Rice
Source: Plants (Basel). 2023 Nov 15;12(22):3859. doi: 10.3390/plants12223859 (PMC10675544; doi:10.3390/plants12223859)
Supplement: Supplementary file 1 [file plants-12-03859-s001.zip › Figure S1-S3.pdf]

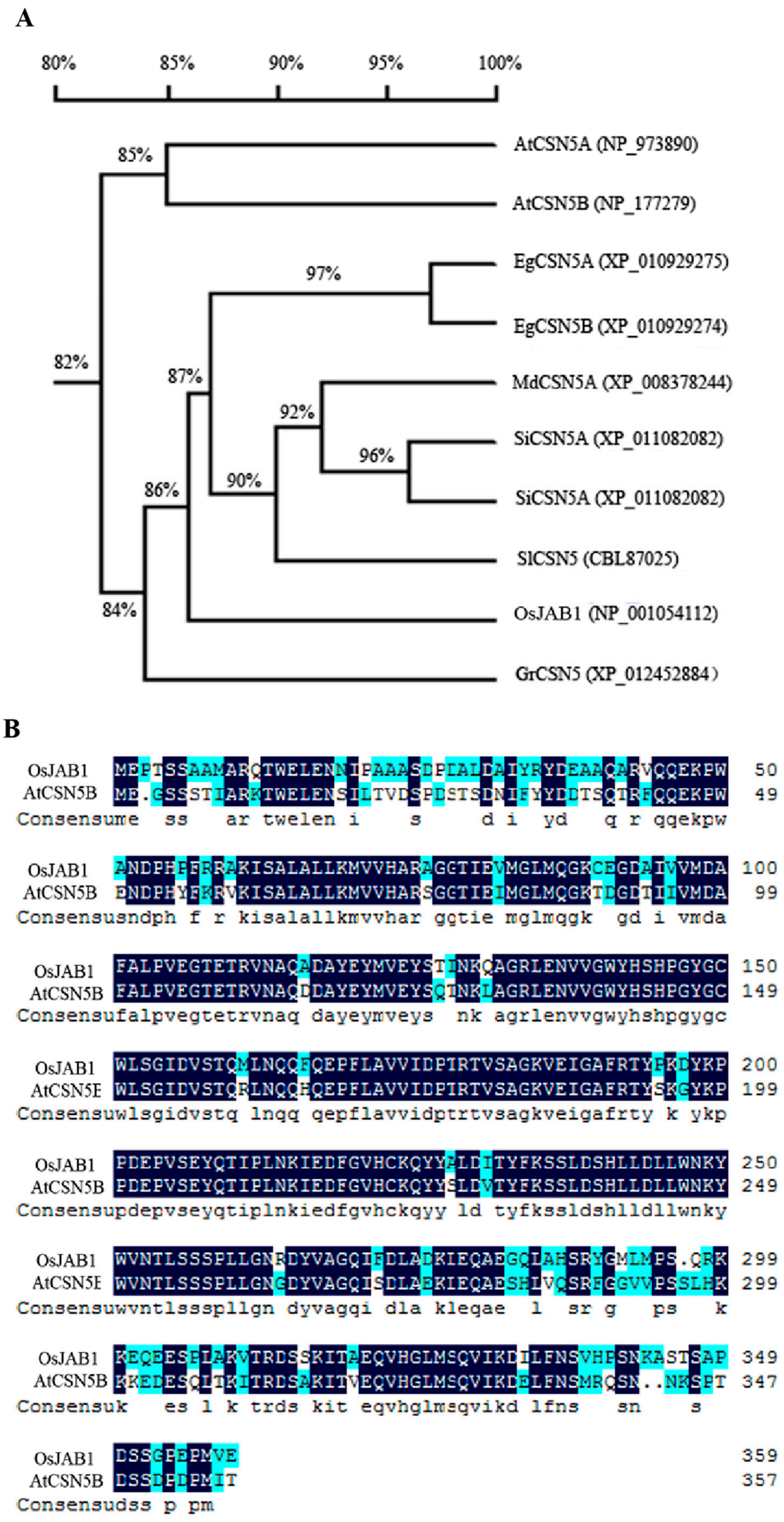
A
80%
85%
90%
95%
100%
AtCSN5A (NP_973890)
AtCSN5B (NP_177279)
EgCSN5A (XP_010929275)
EgCSN5B (XP_010929274)
MdCSN5A (XP_008378244)
SiCSN5A (XP_011082082)
SiCSN5A (XP_011082082)
SlCSN5 (CBL87025)
OsJAB1 (NP_001054112)
GrCSN5 (XP_012452884)
85%
97%
82%
87%
92%
96%
90%
86%
84%
B
OsJAB1 MEPTSSAAMARQTWELENNIPAAASDPLALDAIYRYDEAACARVCQQEKPW 50
AtCSN5B ME.GSSSTIARKTWELENSILTVDSPDSTSDNIFYYDDTSQTRFCQQEKPW 49
Consensume ss ar twelen i s d i yd q r qqekpw
OsJAB1 ANDPHPERRAKISALALLKMVVHARAGGTIEVMGLMQGKCEGDAIVVMDA 100
AtCSN5B ENDPHYFKRVKISALALLKMVVHARSGGTIEIMGLMQGKTDGDTIIVMDA 99
Consensusndph f r kisalallkmvvhar ggtie mglmqgk gd i vmda
OsJAB1 FALPVEGTETRVNAQADAYEYMVEYSTINKQAGRLENVVGWYHSHPGYGC 150
AtCSN5B FALPVEGTETRVNAQDDAYEYMVEYSQTNKLAGRLENVVGWYHSHPGYGC 149
Consensufalpvegtetrvnaq dayeymveys nk agrlenvvgwyhshpgygc
OsJAB1 WLSGIDVSTQMLNQQFQEPFLAVVIDPTRTVSAGKVEIGAFRTYPKDYKP 200
AtCSN5E WLSGIDVSTQRLNQQHQEPFLAVVIDPTRTVSAGKVEIGAFRTYSKGYKP 199
Consensuwlsgidvstq lnqq qepflavvidptrtvsagkveigafrty k ykp
OsJAB1 PDEPVSEYQTIPLNKIEDFGVHCKQYYALDITYFKSSLDSHLLDLLWNKY 250
AtCSN5B PDEPVSEYQTIPLNKIEDFGVHCKQYYSLDVTYFKSSLDSHLLDLLWNKY 249
Consensupdepvseyqtiplnkiedfgvhckqyy ld tyfkssldshlldllwnky
OsJAB1 WVNTLSSSPLLGNRDYVAGQIFDLADKIEQAEGQLAHSRYGMLMPS.QRK 299
AtCSN5E WVNTLSSSPLLGNGDYVAGQISDLAEKIEQAESHIVQSRFGGVVPSSLHK 299
Consensuwvntlssspllgn dyvagqi dla kieqae l sr g ps k
OsJAB1 KEQEESPLAKVTRDSSKITAEQVHGLMSQVIKDILFNSVHPSNKASTSAP 349
AtCSN5B KKEDESQLTKITRDSAKITVEQVHGLMSQVIKDELFNSMRQSN..NKSPT 347
Consensuk es l k trds kit eqvhglmsqvikd lfns sn s
OsJAB1 DSSGEEPMVE 359
AtCSN5B DSSDEDPMIT 357
Consensudss p pm

Figure S1: Phylogenetic analysis of *Arabidopsis* CSN5B homologous proteins and alignment of OsJAB1 and CSN5B. (A) Phylogenetic analysis of OsJAB1 homologous proteins in different plant species. The phylogeny was analyzed using the Blastp search program of the National Center for Biotechnology Information (NCBI, http://www.ncbi.nlm.nih.gov/, accessed on 13 March 2021). (B) The alignment of OsJAB1 and CSN5B proteins and different colors represent different similarity

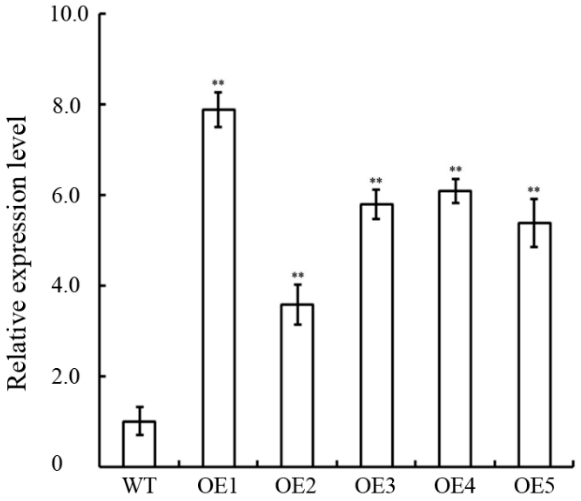

Figure S2: The expression levels of *OsJAB1* in four-week-old seedlings of OEs compared to those in WT. The expression level of *OsActin1* was used as an internal control. Bars represent the SE (±) from three repeated experiments. Asterisks show the significant differences evaluated using *t*-tests (** $p < 0.01$).

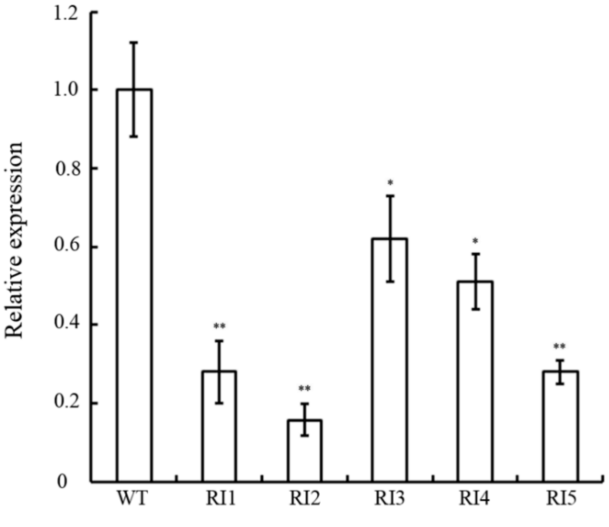

Figure S3: The expression levels of *OsJAB1* in four-week-old seedlings of RIs compared to those in WT. The expression of *OsActin1* was used as an internal control. Bars represent the SE (±) from three repeated experiments. Asterisks represent the significant differences as evaluated using *t*-tests (* $p < 0.05$ and ** $p < 0.01$).
