# Supplementary material for: OsJAB1 Positively Regulates Ascorbate Biosynthesis and Negatively Regulates Salt Tolerance Due to Inhibiting Early-Stage Salt-Induced ROS Accumulation in Rice
Source: Plants (Basel). 2023 Nov 15;12(22):3859. doi: 10.3390/plants12223859 (PMC10675544; doi:10.3390/plants12223859)
Supplement: Supplementary file 1 [file plants-12-03859-s001.zip › Table S1.pdf]

Table S1. Primers used in quantitative real-time PCR

Gene name	Forward primer (5′-3′)	Reverse primer (5′-3′)
OsJAB1 (Os04g0654700)	GGTAACTCGGGATAGCTC	TGCTTCAACCATAGGCTCAG
OsActin (Os02g0704000)	TGGCATCTCTCAGCACATTCC	TGCACAATGGATGGGTCAGA
OsAPX1 (LOC_Os03g17690)	GTGACAAGGAGGGCCTTCTTCA	ATCAGCGAACCCCAGTTCGGA
OsAPX2 (LOC_Os07g49400)	CCGAGCTTGTGAGTGGCGAGA	CAGCAAATCCCAGTTCAGAGA
OsGR3 (LOC_Os10g28000)	CAACAGACAGATATCCGTA	TACTATCAACATCCTGAAGC
OsDHAR1 (AY074786)	CAGAACATTTCAGCTGCTGAC	GGCTAAACAGAGCCTCTGTGTA
OsCAT3 (GU248529)	GCACCAGACAGACAAGAACGA	GCCTCACATTCATACGAGA
