# Supplementary figures and images for: OsJAB1 Positively Regulates Ascorbate Biosynthesis and Negatively Regulates Salt Tolerance Due to Inhibiting Early-Stage Salt-Induced ROS Accumulation in Rice
Source: Plants (Basel). 2023 Nov 15;12(22):3859. doi: 10.3390/plants12223859 (PMC10675544; doi:10.3390/plants12223859)

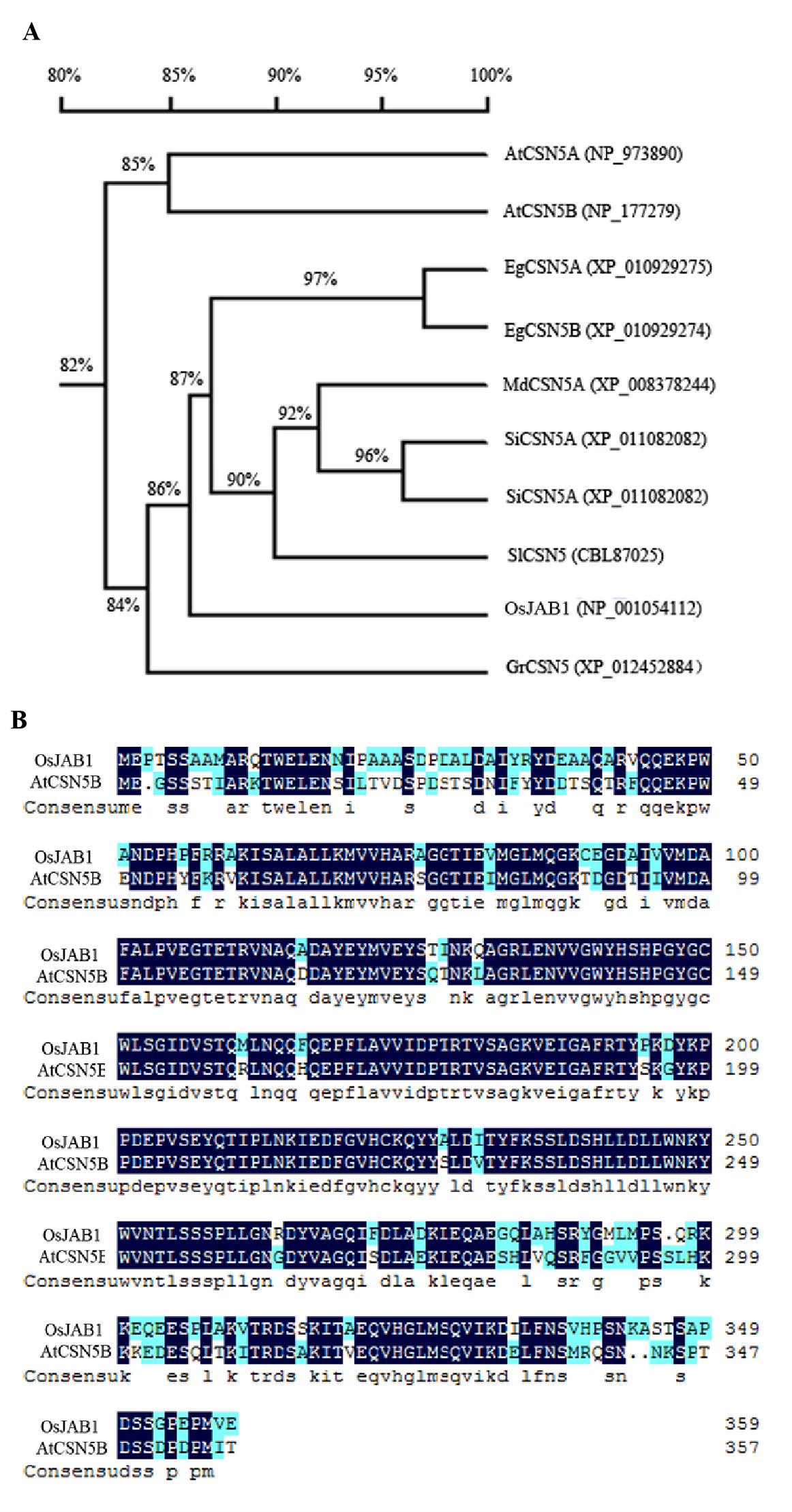

Supplement: Supplementary file 1 [file plants-12-03859-s001.zip › Fig S1.tif]

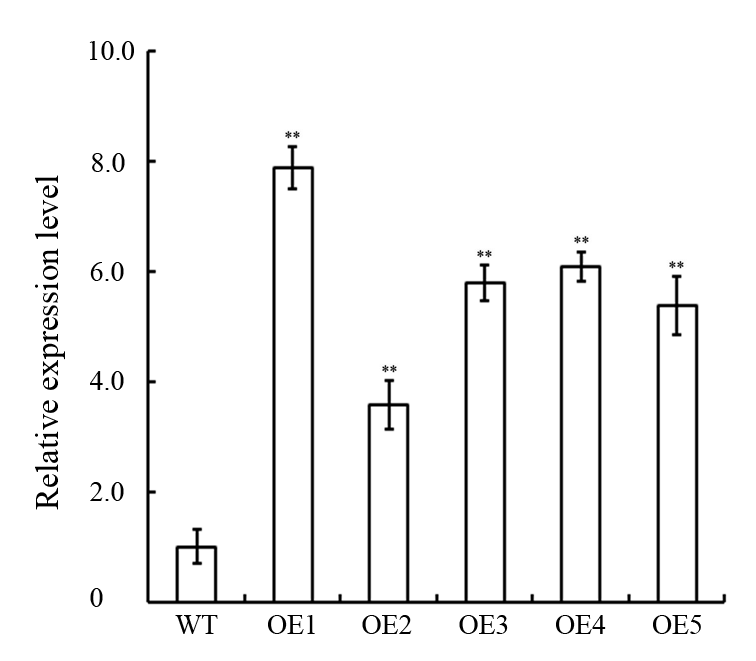

Supplement: Supplementary file 1 [file plants-12-03859-s001.zip › Fig S2.tif]

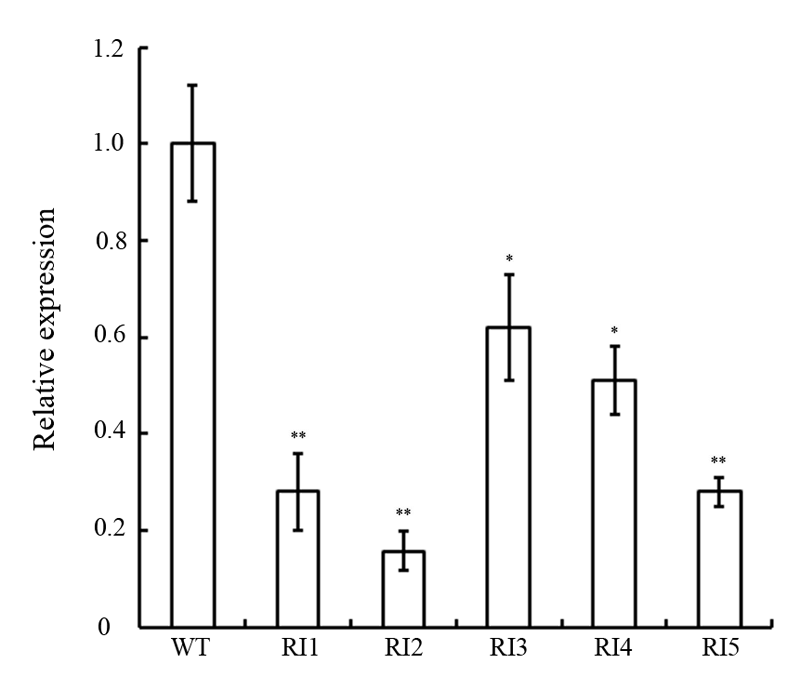

Supplement: Supplementary file 1 [file plants-12-03859-s001.zip › Fig S3.tif]
